# Vitamin B_12_ and Folate Markers Are Associated with Insulin Resistance During the Third Trimester of Pregnancy in South Asian Women, Living in the United Kingdom, with Gestational Diabetes and Normal Glucose Tolerance

**DOI:** 10.1093/jn/nxab352

**Published:** 2021-11-09

**Authors:** Agata Sobczyńska-Malefora, Chittaranjan S Yajnik, Dominic J Harrington, Graham A Hitman, Sarah Finer

**Affiliations:** Nutristasis Unit, Viapath, St. Thomas’ Hospital, London, United Kingdom; Faculty of Life Sciences & Medicine, King's College London, London, United Kingdom; Diabetes Unit, KEM Hospital, Pune, India; Nutristasis Unit, Viapath, St. Thomas’ Hospital, London, United Kingdom; Faculty of Life Sciences & Medicine, King's College London, London, United Kingdom; Blizard Institute, Queen Mary University of London, London, United Kingdom; Wolfson Institute of Population Health, Queen Mary University of London, London, United Kingdom; Barts Health NHS Trust, London, United Kingdom

**Keywords:** pregnancy, gestational diabetes, insulin resistance, vitamin B_12_, folate

## Abstract

**Background:**

Gestational diabetes mellitus (GDM) can adversely affect the health of the developing fetus. Women of South Asian origin are particularly at risk of developing GDM. Insulin resistance (IR) contributes to the etiology of GDM, and although studies have shown associations of vitamin B_12_ (B_12_) and folate status with GDM and IR, only a limited number of B_12_ and folate markers have been used.

**Objective:**

We used a comprehensive panel of B_12_ and folate markers to examine their association with IR in pregnant women with diet-controlled GDM and normal glucose tolerance (NGT).

**Methods:**

In this cross-sectional study, 59 British-Bangladeshi women (24 GDM and 35 NGT) with a mean age of 29 y, BMI (in kg/m^2^) 26.7 and gestational age 33 wk were recruited. Serum total B_12_, holotranscobalamin, folate, methylmalonic acid, plasma homocysteine, 5-methyltetrahydrofolate, and red cell folate (RCF) were measured along with other parameters. The independent sample *t*-test and chi-squared test were used to assess differences in markers between GDM and NGT women. Spearman's test was used to look for correlations. A simple multiple regression analysis was used to investigate if markers of B_12_ and folate status predicted IR, using the HOMA-IR and adjusting for age, GDM status, and BMI.

**Results:**

There were no differences in concentrations of B_12_ and folate markers between GDM and NGT women. In Spearman's analysis HOMA-IR correlated negatively with total serum B_12_ (*P* < 0.001) and holotranscobalamin (*P* < 0.05), and positively with BMI (*P* < 0.001), blood pressure (*P* < 0.05) and triglycerides (*P* < 0.05) in all women. MMA did not correlate with any of the B_12_ markers. In regression analysis, total B_12_ (*β* = −0.622, *P* = 0.004), RCF (*β* = 0.387, *P* = 0.018), and BMI (*β* = 0.024, *P* < 0.001) were the significant predictors of HOMA-IR variance.

**Conclusions:**

Significant associations between markers of B_12_ and folate status with HOMA-IR were found during the third trimester in British-Bangladeshi women. B_12_ markers correlated poorly with each other.

## Introduction

A healthy maternal environment is critical to fetal development. Gestational diabetes mellitus (GDM) (glucose intolerance in pregnancy) is one of the most common disorders of pregnancy. Insulin resistance (IR) is thought to contribute to the etiology of GDM. Pregnancy naturally predisposes to IR as a result of the physiological adaptation necessary to provide glucose to the growing fetus. In early pregnancy reduced IR promotes maternal adipose tissue accumulation. As pregnancy progresses increased maternal IR helps nutrient transfer to promote fetal growth (1). To compensate for IR, there is increased production of insulin from pancreatic beta cells. In normal pregnancies, there is an approximate 50% decrease in insulin-mediated glucose disposal in late pregnancy and a 200% to 250% increase in insulin secretion to maintain euglycemia in the mother (2). GDM results from a reduced capacity for insulin production, and it has been suggested that women who develop GDM have pre-existing defects in insulin action and secretion and are, therefore, predisposed to developing type 2 diabetes (3, 4).

A family history of diabetes, BMI (in kg/m^2^) >30, previous GDM, a previous macrosomic baby weighing ≥4.5 kg, and ethnicity are common clinical risk factors for developing GDM. Women of South Asian origin are particularly at risk of developing GDM due to genetic, intrauterine, socioeconomic, and behavioral risk factors. In some Asian countries the prevalence of GDM reaches 25% compared with ∼10% in North America (5, 6). GDM is also associated with adverse effects on the fetus, e.g., macrosomia, neonatal hypoglycemia, and long-term risk of adiposity and type 2 diabetes via fetal programming (7–11).

Lower muscle mass, higher percentage of body fat and higher IR compared with other populations are the characteristic phenotypic features which have been observed in Asians, as early as at birth (12). Intrauterine undernutrition during pregnancy was suggested as a contributing factor to this phenotype (12). A study conducted in India found that higher maternal folate concentrations during pregnancy predicted greater adiposity and higher IR in the offspring, and lower vitamin B_12_ (B_12_) concentrations predicted higher IR (13). Children born to mothers with high folate but low B_12_ concentrations were the most insulin resistant (13). Other studies have also suggested that excessive use of folic acid (commonly taken as a pregnancy supplement) may adversely affect the health of the fetus, especially in combination with low B_12_, and that this may be additionally associated with GDM risk and IR (13–17). The mechanisms behind this relation are currently unknown but could include a functional folate deficiency through the methyl-trap phenomenon, leading to DNA hypomethylation, impaired metabolism of homocysteine causing endothelial dysfunction, and oxidative stress (5). Deficiency of B_12_ also impacts transfer of fatty acids into mitochondria, resulting in impaired beta-oxidation. This would promote lipo- and adipogenesis and consequently increased risk of IR (5, 18).

To date, B_12_ and folate status in the studies linking IR and GDM with B_12_ and folate status have predominately been assessed using serum total B_12_ or folate measurements while applying non–pregnancy derived cutoffs for the interpretation of these markers. Serum total B_12_ in particular has been criticized as a marker owing to its lack of sensitivity to B_12_ deficiency (19), and it is difficult to interpret this marker because serum B_12_ concentrations decrease during pregnancy because of hemodilution and decreased synthesis of haptocorrin (the most abundant B_12_ binding protein). The functional markers of B_12_ deficiency, serum methylmalonic acid (MMA) and total plasma homocysteine (Hcy), are also affected in pregnancy (20). Holotranscobalamin (HoloTC), the biologically active fraction of B_12_, is thought to be less impacted (21).

The primary aim of this work was to evaluate associations of B_12_ and folate status with IR using a comprehensive panel of markers during the third trimester of pregnancy in British-Bangladeshi women with normal glucose tolerance (NGT) and GDM. To evaluate B_12_ status we used serum total B_12_, serum HoloTC, serum MMA, plasma Hcy, and a combined indicator of B_12_ status (cB_12_); and for folate status we used serum and red cell folate (RCF) and plasma 5-methyltetrahydrofolate (5-MTHF). We also set out to evaluate B_12_ and folate status using the typical non–pregnancy related cutoffs which are routinely applied to pregnant women in the UK. This investigation forms a secondary analysis in a study designed to investigate fetal epigenetic differences associated with maternal gestational diabetes (8).

## Methods

### Subjects

Pregnant British-Bangladeshi women with and without GDM, attending the antenatal care unit at the Royal London Hospital, London, United Kingdom, were recruited during the third trimester of pregnancy (8). The diagnosis of GDM was made based on a 2-h 75-g oral glucose tolerance test (OGTT) following an 8-h fast, using a standard clinical protocol, as part of routine antenatal care at 28 wk of gestation. Blood sampling was taken at 0 and 120 min post glucose ingestion. Local diagnostic cutoffs were used for GDM diagnosis (0-min serum glucose ≥5.8 and/or 120-min ≥7.8 mmol/L). Women with multiple pregnancies or pre-existing diabetes, as well as women who were taking metformin and/or were on insulin therapy, were excluded from the analysis.

At recruitment, the women were asked about iron, folic acid, and multivitamin usage during pregnancy, and based on their responses we grouped these subjects into iron, folic acid, or multivitamin users.

Blood samples were taken to assess folate and B_12_ status, total cholesterol, LDL cholesterol, HDL cholesterol, and triglycerides (TG). Blood pressure (BP) was also recorded.

Pregnancy notes were used to collect anthropometric data in the first trimester. Pregnancy history was recorded. Neonatal measurements included the following: weight, height, head circumference (HC), chest circumference (CC), and abdominal circumference. The study was approved by the NHS Research Ethics Committee (Ref 09/H0718/31; IRAS ID 10031).

### Analytical methods

Fasting blood samples (30 ml) following an 8-h fast were collected from each woman after a diagnosis toward GDM had been made and standard dietary management had been initiated (22). Blood samples were placed on ice, and whole blood was separated into serum/plasma within 2 h of collection using standard procedures. Serum/plasma samples were stored at −80°C until analysis. Fasting insulin was measured by an electrochemiluminescent sandwich immunoassay (Roche) and serum glucose (as part of an oral glucose tolerance test) by hexokinase assay (Roche).

Serum cholesterol, TG, and LDL-, and HDL cholesterol were measured on Cobas 6000 (Roche) with satisfactory analytical performance monitored through use of the UKNEQAS, external quality assurance (EQA) scheme.

Serum folate and total serum B_12_ were performed by an electrochemiluminescent competitive immunoassay (Roche), whereas a competitive binding paramagnetic particle assay (Beckman Coulter) was used for RCF. These tests were performed by Barth Health NHS Trust, London, United Kingdom, with satisfactory analytical performance monitored through use of the UKNEQAS EQA scheme. We applied the standard reference ranges used at Barts Health NHS for these tests as these are routinely applied to all subjects, including pregnant women. The reference ranges are: 8.6–45.3 nmol/L for serum folate, 140–664 pmol/L for serum total B_12_, and 362–1450 nmol/L for RCF.

Serum Hcy was measured by HPLC at Homerton NHS Trust, EQA scheme UKNEQAS; cutoff for pregnancy <10 μmol/L. Serum HoloTC and MMA and plasma 5-MTHF were measured at the Nutristasis Unit, Viapath, St. Thomas’ Hospital. Serum HoloTC analysis was carried out using an Architect 2000 (Abbott Diagnostics). For 5-MTHF, an HPLC assay was utilized as described elsewhere (20, 23). LC-MS/MS was used for the determination of MMA (Gerstel Multi-Purpose Sampler coupled directly to Agilent Technologies 6460 Triple Quad) (24). External quality assurance schemes were used for all assays; UKNEQAS for HoloTC and 5-MTHF and DEKS for MMA. Vitamin B_12_ deficiency is suggested if HoloTC is <25 pmol/L and MMA >280 nmol/L, whereas folate deficiency is suggested if plasma 5-MTHF is <7.6 nmol/L (23, 25).

A combined indicator of B_12_ status (cB12) was calculated using a set of formulas which combine the results of a panel of B_12_ status markers, i.e., cB_12_ = log_10_ (HoloTC × B12/MMA × Hcy)_test_ − log_10_ (HoloTC × B12/MMA × Hcy)_ref_ = test − 3.79/1 + (age/230)^2.6^ (26). Reference combinations were derived from a database (*n* = 5211) following mathematical modeling. cB12 has been suggested to be a more reliable indicator of B_12_ status than individual tests. Depending on the cB12 value obtained, B_12_ status is classified as: elevated (>1.5), adequate (0.5–1.5), low (−1.5 to −0.5), possible B_12_ deficiency (−2.5 to −1.5), or probable B_12_ deficiency (<−2.5).

HOMA-IR, which is derived from a mathematical assessment of the balance between hepatic glucose output and insulin secretion, was calculated using fasting glucose and insulin concentrations (27, 28). Increased values of HOMA-IR are indicative of IR.

### Statistical analysis

The Shapiro–Wilk test was used to check the normality of data. Serum total B_12_, HoloTC, and folate and plasma 5-MTHF and RCF results were inspected for outliers using the outlying labeling rule, g = 2.2 (29), to identify women on potentially very high B_12_ or folate supplements. As a result of these analyses, 1 serum B_12_, 4 HoloTC, 1 RCF, and 1 5-MTHF result were excluded from the analyses. Where applicable, results not normally distributed were log transformed before analysis. We used descriptive statistics to summarize characteristics of women with NGT and GDM, including their B_12_ and folate status, and applied the Wilcoxon signed rank test, independent sample *t*-test, and chi-squares tests (as appropriate) to test for differences between women with NGT and with GDM, and the selected baby parameters from babies born to NGT and GDM mothers. Spearman's test was used to investigate correlations, first between HOMA-IR with other variables measured and second between B_12_ and folate markers. Multiple regression analyses was used to study independent predictors of HOMA-IR from the selected variables: age, gestational age, GDM status, BMI, diastolic pressure, serum total B_12_, serum HoloTC, serum MMA, plasma Hcy, RCF, serum folate, and plasma 5-MTHF. Due to multicollinearity between B_12_ and folate markers, separate models were constructed by adding serum B_12_ and HoloTC and serum folate, 5-MTHF, RCF, and Hcy separately. Statistical analyses were carried out using SPSS, v. 26.

## Results

### Characteristics of the study population

Compared with women without GDM, women with GDM were older (*P* = 0.035), had a higher gestational age at the time of testing (*P* = 0.035), and had higher glucose concentrations during OGTT (**Table 1**). No other measurements related to adiposity or diabetic predisposition differed between the 2 groups (Table 1).

**TABLE 1:** Characteristics of the study cohort and comparison of women with NGT compared with women with GDM^1^

Variable	All	NGT	GDM	*P* value
*n* (%)	59 (100)	35 (60)	24 (40)	
Age, y	29.0 ± 5.2	27.7 ± 4.8	30.8 ± 5.2	0.035
Height, m	1.56 ± 0.57	1.56 ± 0.58	1.55 ± 0.56	0.448
Weight,^2^ kg	60 (53–72)	60 (52–75)	60 (54–66)	0.660
BMI,^2^ kg/m^2^	26.7 ± 5.5	27.3 ± 6.4	25.7 ± 3.8	0.579
BMI 23.0–27.5 kg/m^2^, *n* (%)^3^	15 (25)	8 (23)	7 (29)	0.45
BMI >27.5 kg/m^2^, *n* (%)^3^	22 (37)	13 (37)	9 (37)	0.487
Parity, *n*	1.6 ± 1.5	1.6 ± 1.5	1.8 ± 1.5	0.329
Serum OGTT 0 h, mmol/L	4.6 (4.2–5.0)	4.5 (4.3–5.0)	4.7 (4.2–5.3)	0.289
Serum OGTT 2 h, mmol/L	6.9 (5.4–8.5)	5.5 (4.9–6.6)	9.0 (8.0–9.6)	<0.001
Gestational age at blood sampling, wk	33 (30–35)	32 (29–34)	34 (32–35)	0.035
Serum fasting glucose, mmol/L	4.4 (4.1–5.0)	4.4 (4.2–5.1)	4.3 (4.0–4.8)	0.516
Serum fasting insulin, mIU/L	13.4 (9.1–21.9)	13.3 (9.2–24.5)	13.6 (8.9–18.4)	0.807
HOMA-IR	1.58 (1.09–2.38)	1.61 (1.18–2.90)	1.69 (1.06–2.26)	0.641
Serum LDL-C, mmol/L	3.6 (2.9–4.7)^4^	3.41 (2.8–4.9)^5^	3.72 (3.0–4.1)^6^	0.863
Serum TC, mmol/L	5.9 (5.0–7.0)^7^	5.9 (5.0–7.1)^8^	5.9 (5.1–6.4)^6^	0.906
Serum TG, mmol/L	2.3 (1.7–3.0)^7^	2.4 (1.5–3.0)^8^	2.1 (2.0–2.8)^6^	1.000
Serum HDL-C, mmol/L	1.4 ± 0.3^7^	1.4 ± 0.3^8^	1.5 ± 0.3^6^	0.756
Systolic BP, mmHg	110 ± 11	111 ± 13	107 ± 8	0.237
Diastolic BP, mmHg	70 ± 11	70 ± 11	70 ± 10	0.865

1 Values are shown as mean ± SD or median (IQR) unless otherwise indicated. BP, blood pressure; C, cholesterol; FH, family history; GDM, gestational diabetes mellitus; NGT, normal glucose tolerance; OGTT, oral glucose tolerance test; TC, total cholesterol; TG, total triglycerides.

2 First trimester.

3 WHO recommended BMI cutoffs for Asian people: 23.0–27.5 kg/m^2^ increased cardiovascular and diabetic risk, >27.5 kg/m^2^ higher high risk (56).

4 *n* = 41.

5 *n* = 24.

6 *n* = 17.

7 *n* = 39.

8 *n* = 22.

### Vitamin status and the prevalence of deficiencies/high status based on routinely-used cutoffs

There were no differences in the concentrations of B_12_ and folate markers between NGT and GDM women (**Table 2**). The numbers of women taking iron supplements and folic acid were similar in the 2 groups but there was an excess of multivitamin supplement users in the GDM group (*P* = 0.039).

**TABLE 2:** Number of vitamin/supplement users and concentrations of markers related to vitamin status^1^

Variable	All	NGT	GDM	*P* value
Multivitamin users,^2^ *n* (%)	37 (63)	21 (60)	16 (67)	0.039
Folic acid, *n* (%)	52 (88)	30 (86)	22 (92)	0.561
Iron supplement, *n* (%)	44 (75)	24 (67)	20 (83)	0.505
Serum total B_12_, pmol/L (*n* = 57)	188 (142–247)	192 (132–244)	169 (142–206)	0.308
Serum HoloTC, pmol/L (*n* = 55)	51.0 (37.4–65.0)	49.0 (37.0–60.0)	51.8 (38.5–84.0)	0.291
Serum folate, nmol/L (*n* = 59)	16.9 (12.2–31.3)	16.7 (12.2–28.4)	16.0 (12.1–25.4)	0.899
Plasma 5-MTHF, nmol/L (*n* = 58)	16 (9.8–27.3)	15.7 (10.1–27.8)	15.1 (9.1–23.7)	0.628
RCF, nmol/L (*n* = 55)	713 (532–996)	684 (496–791)	722 (552–869)	0.260
Plasma Hcy, μmol/L (*n* = 58)	4.9 (4.1–6.4)	4.9 (4.4–6.3)	5.7 (4.6–7.5)	0.224
Serum MMA, nmol/L (*n* = 59)	189 (131–343)	164 (130–350)	244 (149–358)	0.371
cB12 (*n* = 55)	0.407 ± 0.495	0.148 ± 0.504	0.152 ± 0.190	0.963

1 Values are shown as mean ± SD or median (IQR) unless otherwise indicated. cB12, combined indicator of vitamin B_12_ status; Hcy, plasma homocysteine; HoloTC, holotranscobalamin; MMA, methylmalonic acid; RCF, red cell folate; 5-MTHF, 5-methyltetrahydrofolate.

2 24% of women were taking Healthy Start tablets. The daily dose in 1 tablet contained 70 mg of vitamin C, 10 μg of vitamin D, 400 μg of folic acid.

Combined analysis of the whole group (*n* = 59) (Table 2) showed variations in estimation of the prevalence of B_12_ deficiency when using individual markers and applying routinely used nonpregnant reference ranges for most markers: 21% with serum total B_12_ (<140 pmol/L), 3% with HoloTC (<25 pmol/L), 5% with serum Hcy (>10 μmol/L; suggested for pregnancy), and 31% with serum MMA (>280 nmol/L) (25, 30). Only 5% of women had folate deficiency as estimated by serum folate (<8.6 nmol/L) or RCF (<362 nmol/L), whereas 14% of women had low plasma 5-MTHF (<7.6 nmol/L) (23, 31). Five percent of women had high serum folate (>45.3 nmol/L), 7% had high RCF (>1450 nmol/L) and 20% had high plasma 5-MTHF (>42.0 nmol/L) (32). There was no difference in the prevalence of B_12_ and folate deficiency or high folate concentrations between NGT and GDM women (data not shown).

### Examining the association of comprehensive B12 and folate markers with HOMA-IR

Bivariate correlations (Spearman's test) of fasting glucose and fasting insulin with B_12_ and folate markers showed that fasting glucose correlated negatively with HoloTC alone *(ρ* = −0.272, *P* = 0.037), and fasting insulin correlated negatively with serum total B_12_ (*ρ* = −0.402, *P* = 0.002) and HoloTC (*ρ* = −0.306, *P* = 0.020) but not with MMA, cB12, Hcy, and folate markers. HOMA-IR correlated negatively with serum total B_12_ and HoloTC and positively with BMI, BP, and TG in all women (**Table 3**). In a multiple regression analysis, serum total B_12_, RCF, and BMI were the only significant predictors of HOMA-IR variance (**Table 4**).

**TABLE 3:** Selected correlation coefficients between HOMA-IR and other variables measured^1^

Variable	*ρ* ^2^
Age, y	0.074
Gestation period of blood sample, wk	0.073
Fasting glucose, mmol/L	0.438**
Fasting insulin, mIU/L	0.996***
BMI, kg/m^2^	0.494***
Vitamin use	0.034
Smoking	0.199
Serum total B_12_, pmol/L	−0.443***
Serum HoloTC, pmol/L	−0.319*
Serum MMA, nmol/L	0.024
cB12	−0.194
Serum folate, nmol/L	−0.077
Plasma 5-MTHF, nmol/L	−0.029
RCF, nmol/L	0.129
Plasma Hcy, μmol/L	−0.060
Systolic BP, mmHg	0.286*
Diastolic BP, mmHg	0.293*
LDL-C, mmol/L	−0.107
Serum TC, mmol/L	−0.058
Serum TG, mmol/L	0.36*
HDL-C, mmol/L	−0.098

1 All women, *n* = 59. BP, blood pressure; C, cholesterol; cB12, combined indicator of vitamin B_12_ status; FH, family history; Hcy, plasma homocysteine; HoloTC, holotranscobalamin; MMA, methylmalonic acid; OGTT, oral glucose tolerance test; RCF, red cell folate; TC, total cholesterol; TG, total triglycerides.

2 Values are Spearman's rank corelations coefficient *ρ*. ^*, **, ***^Significant correlation: *P < 0.05, **P < 0.01, ***P < 0.0001.

**TABLE 4:** Multiple regression analyses in the whole group to demonstrate the relation between serum total B_12_, RCF, and other potential variables and HOMA-IR^1^

	*R* ^2^ = 0.405		
HOMA-IR^2^	*β*	95% CI for *β*	*P* value
Age	−0.005	(−0.017, 0.008)	0.459
Gestational age, wk	<0.001	(−0.024, 0.023)	0.968
BMI, kg/m^2^	0.024	(0.012, 0.036)	<0.001
GDM status	−0.064	(−0.194, 0.066)	0.323
Diastolic BP, mmHg	0.002	(−0.004, 0.009)	0.467
Serum total B_12_,^2^ pmol/L	−0.622	(−1.049,−0.195)	0.005
Serum MMA,^2^ nmol/L	−0.070	(−0.266, 0.127)	0.477
RCF,^2^ nmol/L	0.387	(0.070, 0.704)	0.018

1 *n* = 54. BP, blood pressure; GDM, gestational diabetes mellitus; Hcy, plasma homocysteine; MMA, methylmalonic acid; RCF, red cell folate.

2 Transformed to log values for statistical calculations. *R*^2^, adjusted; *β*, unstandardized coefficients.

In another multiple regression model with HoloTC as a variable instead of serum total B_12_, HoloTC did not have a statistically significant association with HOMA-IR (*P* = 0.197). Serum folate (*P* = 0.352), plasma 5-MTHF (*P* = 0.174), and Hcy (*P* = 0.145) were also not significant predictors of HOMA-IR when added separately to the models (data not shown). Lipids were not included in multiple regressions models because of high percentages of missing data.

### Bivariate correlations between markers of B_12_ and folate status

Since our bivariate correlations and multiple regression analysis showed consistently negative associations between serum total B_12_ (and HoloTC in the correlation) and HOMA-IR, but the same was not seen for MMA and Hcy, bivariate correlations between all B_12_ markers were carried out to see if the expected relations between these markers were present (**Table 5**).

**TABLE 5:** Correlation between vitamin B_12_ markers for the whole group^1^

Variable	*ρ*	*P*
Serum MMA, nmol/L		
Serum B_12_	−0.139	0.299
HoloTC	−0.208	0.113
Hcy	0.032	0.813
cB12	−0.716	<0.001
Serum total B_12_, pmol/L		
HoloTC	0.509	<0.001
Hcy	−0.111	0.41
cB12	0.548	<0.001
Serum HoloTC, pmol/L		
Hcy	−0.312	0.017
cB12	0.707	<0.001
Plasma Hcy, μmol/L		
cB12	−0.432	0.001

1 Values are Spearman's rank correlations coefficient *ρ, n* = 59. cB12, combined indicator of vitamin B_12_ status; Hcy, plasma homocysteine; HoloTC, holotranscobalamin; ; MMA, methylmalonic acid.

Interestingly, MMA did not correlate with serum total B_12_, HoloTC, or Hcy (Table 5). Serum total B_12_ correlated strongly with HoloTC but it did not correlate with Hcy. HoloTC correlated with Hcy in the whole group. Out of 18 women with MMA >280 nmol/L (above the non–pregnancy related cutoff), only 3 (17%) had serum total B_12_ <140 pmol/L, 1 had HoloTC <25 pmol/L and 9 (50%) had a BMI > 27.5.

All folate markers: total serum, plasma 5-MTHF, and RCF correlated well with each other (*ρ* = 0.778–0.981, *P* < 0.001).

### Association of maternal variables with neonatal anthropometry

The mean ± SD values for infant birth weight, head circumference (HC), chest circumference (CC), abdominal circumference (AC), and length were 3.1 ± 0.4 kg, 33.7 ± 1.6 cm, 32.8 ± 1.6 cm, 30.8 ± 2.1 cm, and 47.2 ± 1.9 cm, respectively. Gestational age and anthropometric measurements were similar in the babies of GDM and NGT mothers.

In bivariate analysis, maternal HOMA-IR correlated with baby's length (*ρ* = 0.344, *P* = 0.046) but not with baby's weight (NGT and GDM combined). HOMA-IR correlated with baby's length in women with GDM (*ρ* = 0.625, *P* = 0.013), but not in women with NGT (*ρ* = 0.111, *P* = 0.661).

## Discussion

We found significant associations between serum total B_12_, HoloTC, and RCF with IR (using the homeostatic model HOMA-IR) in Bangladeshi women during the third trimester of pregnancy. These findings were consistent across bivariate analyses (negative associations for serum total B_12_ and HoloTC) and multivariate analyses (negative associations for serum total B_12_ and positive for RCF). These findings are in agreement with those of other studies which have also reported that lower serum total B_12_ concentrations are associated with a higher HOMA-IR index during the third trimester of pregnancy (14, 33).

Unlike previously reported studies which have shown associations between GDM risk and B_12_ and folate status using total serum B_12_ and folate tests, we used a comprehensive panel of biochemical markers of B_12_ and folate status. Most previous studies had quantified serum total B_12_ only, and it is known that this test in particular is greatly affected by pregnancy because of hormonal and B_12_ binding protein changes (34, 35) in addition to declining B_12_ stores due to transfer to the fetus (20). These factors reduce the diagnostic utility of total B_12_ during pregnancy. Our correlation and regression analyses showed that both serum total B_12_ and HoloTC were negatively associated with HOMA-IR. These results confirm previously reported associations and suggest a possible role for B_12_ in IR/GDM risk. It was also of interest to see the significant variations in the prevalence of B_12_ deficiency, in particular when serum total B_12_ and HoloTC markers only were considered, 21% compared with 3%, respectively, and this indicates that the routinely-used cutoffs that are applied to pregnant women are unhelpful. Previous pregnancy studies suggested that HoloTC may be a better marker of B_12_ status during pregnancy than total B_12_ as it is less affected by hormonal changes and by the decrease in the levels of haptocorrin during pregnancy (21, 36). Interestingly, Fernandez-Costa and Metz (37) reported a sharp increase in HoloTC during the third trimester, whereas Murphy et al. (36) found that after the initial reduction in plasma HoloTC in early pregnancy, HoloTC reached a plateau and remained at a lower level until the end of pregnancy. The authors suggested that compensatory mechanisms operate during pregnancy to ensure a B_12_ supply sufficient to meet increased B_12_ requirements in both the mother and the fetus (36). Thus, use of total serum B_12_ may overestimate prevalence of B_12_ deficiency in pregnancy. Consequently, HoloTC could be a better diagnostic tool for the assessment of B_12_ status during the third trimester of pregnancy than serum total B_12_, as supported by our observation that HoloTC levels correlate with homocysteine in our study.

No association of HOMA-IR with MMA was found, and this is an important finding as this test is often used for confirmation of B_12_ status assessment (25, 38). The prevalence of elevated MMA was 31%, but the usual correlations of MMA with serum total B_12_, HoloTC, and Hcy were not seen in this study. Interestingly, only 3 (17%) of the women with MMA >280 nmol/L had serum total B_12_ <140 pmol/L. This finding could suggest that not only serum total B_12_ but also MMA concentrations may be influenced by factors not related to B_12_ status during the third trimester of pregnancy. Hemodilution and hormonal changes have been likewise proposed as modulators of MMA concentration during pregnancy (36). Methylmalonic acid is a product of mitochondrial hydrolysis of excess methylmalonyl-CoA (MMA-CoA), which is formed from the conversion of odd-chain fatty acids, cholesterol and branched chain amino acids, via propionate. MMA-CoA is converted to succinyl-CoA, a major intermediate of the citric acid cycle, by a mutase with B_12_ serving as a cofactor. The inhibition of this pathway in B_12_ deficiency leads to the accumulation of MMA-CoA, which can inhibit carnitine palmitoyl transferase system, an essential step in the beta-oxidation of long fatty acids, thus promoting lipogenesis and insulin resistance (14, 39, 40). Conversely, the higher use of fats than carbohydrates for energy toward the end of pregnancy may also result in more MMA-CoA being converted to MMA regardless of B_12_ status.

Similarly to MMA, another functional marker of B_12_ deficiency, Hcy, did not show any associations with HOMA-IR and did not correlate with serum total B_12_ nor MMA, but it correlated with HoloTC. Increased Hcy concentrations and their impact on the impairment of endothelial function leading to diabetes (16) have been proposed as another mechanism contributing to GDM risk. However, whilst some studies demonstrated differences in Hcy concentrations between women with NGT and GDM or associations with GDM risk (16) (41–43), others failed to observe the same (17, 44). This is possibly because Hcy concentrations are normally much lower during pregnancy. Hemodilution, hormonal changes, and folic acid supplementation are probably the main reasons for low concentrations of Hcy in pregnancy. Moreover, subnormal Hcy was reported in nondiabetic hyperinsulinemic subjects in the prediabetic stage (45). Glomerular hyperfiltration observed in early diabetes and metabolic effects of high insulin levels have been proposed as mechanisms for low Hcy (46, 47). Therefore, Hcy concentrations during the third trimester may be significantly dependent on metabolic and hormonal changes and thus may not be reliable as a marker of B_12_ or folate status. More evidence is required to demonstrate that higher homocysteine may be associated with higher IR and GDM risk.

cB12 estimation provides a more comprehensive evaluation of B_12_ status as it takes into account all B_12_ markers and makes adjustments for patient age and folate status. So far, cB12 has not been validated in pregnancy. As serum total B_12_, MMA and Hcy in particular are probably affected by other factors independent of B_12_ status during pregnancy, the utility of cB12 as a marker in pregnancy needs further evaluation. As expected, cB12 correlated with all B_12_ markers. The prevalence of low B_12_ status assessed using cB12 was similar to that assessed using HoloTC.

The underlying mechanisms for the associations of HOMA-IR with B_12_ status during the third trimester of pregnancy are currently unknown. Although the role of B_12_ in lipid metabolism has not been fully elucidated, the inhibition of fatty acid oxidation is one of the proposed mechanisms leading to insulin resistance (13). Studies have demonstrated that adipocytes cultured in low B_12_ conditions had increased cholesterol and homocysteine concentrations combined with hypomethylated promoter regions of the key regulator genes (*SREBF1* and the LDR receptor (LDLR)) of cholesterol biosynthesis (48) as well as a significantly increased expression of genes involved in triglyceride biosynthesis and decreased expression of *β*-oxidation genes (49). Other in vitro studies have found that adipocytes in low B_12_ conditions displayed increased lipid accumulation (50). In our study, triglycerides correlated positively with HOMA-IR (Table 3) and negatively with serum total B_12_ (*ρ* = −0.345, *P* = 0.034) (data not shown). Thus, maternal body fat distribution/metabolism during pregnancy may be implicated in determining circulating concentrations of micronutrients in pregnancy as previously suggested (33). As BMI is a strong predictor of HOMA-IR, it will be important for future studies to elucidate whether body fat has a causal role in the relation between B_12_ status and HOMA-IR, or whether this is confounded by other factors such as socioeconomic status and vitamin supplement use. Such an analysis is not possible in a cross-sectional study.

No significant differences were found between women with NGT and GDM with regard to B_12_ status. This was true for all B_12_ markers used, including cB12. This is unlike the results reported by Krishnaveni et al. (14) and Sukumar et al. (15), who identified higher rates of B_12_ deficiency in pregnant women with GDM than those with NGT in South Indian and white European populations, respectively. Results of a recent systematic review and meta-analysis also suggested that pregnant women with B_12_ deficiency are at almost 2-fold higher risk of developing GDM than women with B_12_ sufficiency (51). However, it should be noted that this meta-analysis included only 2 studies (14, 15), which are discussed above. The findings in our study may have been influenced by the small number of participants, and because of this we included women who were taking supplements. However, other studies do concur with our finding that there is no difference in B_12_ status between women with NGT and GDM (17, 44, 52). Future studies examining this relation should examine the direct effects of GDM management on B_12_ and folate status. Current dietary recommendations predominantly focus on carbohydrate restriction and it is conceivable that a reduction in carbohydrate intake could result in replacement by foods which have a greater B_12_ and/or folate content. Although no data were collected showing the supplementation/medication changes after GDM diagnosis, the finding of 4 women with GDM with highly elevated HoloTC (extreme outliers) as well as the higher proportion of women with GDM taking vitamin supplements may support this notion. Moreover, unlike total serum B_12_, HoloTC reflects a more recent intake of B_12_, and GDM diagnosis preceded the sample collection for this study.

In contrary to B_12_ markers, folate markers utilized in this study correlated highly with each other: (*ρ* ≥ 0.8, *P* < 0.001). The prevalence of low and high folate status was higher when 5-MTHF was used as a marker of folate assessment than when serum folate and RCF were used. Methodological differences and cutoff variations, as well as small sample, size may partially account for this finding. The study size did not permit the partition by folate status subgroups incorporating low/normal B_12_ concentrations which could be used to establish if women with low B_12_ and high folate status were more prevalent in the GDM group.

Importantly, RCF was a significant positive predictor of HOMA-IR variance (Table 4). Serum folate and plasma 5-MTHF, included in separate models, did not reach the statistical significance in predicting HOMA-IR variance. RCF is a marker of long-term folate status, whereas serum folate is affected by recent folate intakes (31). Diet control could have had an impact on both serum folate and plasma 5-MTHF. Both low and high serum folate concentrations have been associated with negative health effects (32, 53), and it has been suggested that folate may affect the development of GDM via different pathways (16). More work is needed to address optimum and safe folate supplementation regimes during pregnancy.

The strong associations of HOMA-IR with BMI were also confirmed in this study, both in multivariate and bivariate analysis, as well as the previously reported associations of IR with infant length (54, 55).

## Conclusions

In conclusion, B_12_ and folate status are associated with IR during the third trimester of pregnancy in South Asian women living in the United Kingdom. As the provision of balanced and adequate B_12_ and folate is vital for the wellbeing of both mother and child, markers of status need to be carefully interpreted in view of pregnancy-related changes affecting concentrations of these nutrients. Pregnancy-specific reference ranges should be used for B_12_ markers. Replication of these findings in larger cohorts may provide valuable insights into the mechanisms linking B_12_ and folate status with IR and GDM.

## Notes

Supported by an MRC Clinical Research Training Fellowship (G0800441, awarded to SF), the North-East London Diabetes Research Network and the European Union (FP7 EU grant: 83599025).

Author disclosure: The authors declare no conflict of interest.

Abbreviations used: AC, abdominal circumference; B_12_, vitamin B_12_; BP, blood pressure; cB_12_, combined indicator of vitamin B_12_ status; CC, chest circumference; GDM, gestational diabetes mellitus; HC, head circumference; Hcy, total plasma homocysteine; HoloTC, holotranscobalamin; IR, insulin resistance; MMA, methylmalonic acid; MMA-CoA, methylmalonyl-CoA; NGT, normal glucose tolerance; OGTT, oral glucose tolerance test; RCF, red cell folate; TG, triglycerides; 5-MTHF, 5-methyltetrahydrofolate.
